# Clinical significance of acidic extracellular microenvironment modulated genes

**DOI:** 10.3389/fonc.2024.1380679

**Published:** 2024-09-20

**Authors:** Yasumasa Kato, Kotori Mawatari

**Affiliations:** Department of Oral Function and Molecular Biology, Ohu University School of Dentistry, Koriyama, Japan

**Keywords:** acidic extracellular pH, prognosis, acidosis dependency, pathological staging, cDNA microarray

## Abstract

**Background:**

The extracellular pH (pH*_e_*) is known to be acidic. We investigated the effect of mild (pH*_e_* 6.8) and severe (pH*_e_* 5.9) acidosis on gene expression in mouse B16-BL6 melanoma cells using cDNA microarray analysis and compared them with the acidic pH*_e_* dependence of human tumors.

**Methods:**

B16-BL6 cells were treated with pH*_e_* 7.4 (control), pH*_e_* 6.8, and pH*_e_* 5.9. The mRNA expression was analyzed by using the cDNA microarray. Heat map, volcano plot, and gene ontology enrichment analysis were performed. The data were compared with the gene signatures of published data GSE52031 and GSE8401 and compared with the pathological staging by GEPIA2, and the prognostic signature of proteins was searched by the Human Protein Atlas database. If the acidic pH*_e_*-induced and -reduced genes were correlated with shortened and prolonged survival times, respectively, and also correlated with pathological staging, we defined it as “hit” and counted the sum of hit points of eight types of tumors such as breast, colorectal, prostate, gastric, liver, prostate, lung, and head and neck and melanoma.

**Results:**

Gene expression was differentially and commonly regulated by both pH*_e_* s. The number of genes upregulated fourfold or more at pH*_e_* 6.8 and 5.9 only for 25 and 131 genes, respectively, and 85 genes were common. The number of genes downregulated fourfold or less at pH*_e_* 6.8 and 5.9 only for 63 and 82 genes, respectively, and 118 genes were common. Compared with human mRNA expression data (GSE8401), there is no correlation with the overall pattern of the signature. In seven types of cancer (breast, colorectal, gastric, liver, prostate, lung, and head and neck) and melanoma, the relationship between acidic pH*_e_*-modulated gene expression and overall survival was evaluated. As a result, acidic pH*_e_* dependency contributing to prognosis was higher in colorectal, lung, and head and neck cancers and lower in prostate cancer.

**Conclusion:**

Tumor classification based on response to extracellular acidic pH*_e_* will provide new insights into chemotherapy strategy for patients with tumors.

## Background

It is well known that the extracellular pH (pH*_e_*) in tumor tissue is acidic. Although the Warburg effect (aerobic glycolysis) is undoubtedly the major contributor to tumor extracellular acidity, CO_2_ from the pentose phosphate pathway (PPP) and carbonic anhydrases (CAs), especially CAIX, are also important causes (1). The buffering effect of tumor tissue fluid is weaker than that of normal tissue (2, 3). The acidity of the tumor tissue may contribute to this. Thus, the acidic pH*_e_* acts as a microenvironmental factor on the tumor cells in an autocrine/paracrine manner. In contrast to hypoxia, hypoxia-specific transcription factors such as hypoxia-inducible factor (HIF), the specific transcription factors, have not been identified, and some transcription factors that are common in cytokine signaling, *e.g.*, nuclear factor-κB (NF-κB), have been reported (4–7). In addition, recent studies have shown that acidic pH*_e_* induces signal transducer and activator of transcription 1 (STAT1) (8), and peroxisome proliferator–activated receptor α (PPARα) (9).

Acidic pH*_e_* affects many cellular phenotypes such as epithelial–mesenchymal transition (EMT), angiogenesis, exosome secretion, invasion, and metastasis (10, 11). It has been suggested that acidic pH*_e_* broadly affects the expression of many genes directing more malignant phenotypes such as invasion and metastasis whose activity was well relevant to clinical cases (10–13). The chronic effect of acidosis has also been studied. Acidic pH*_e_*-adapted squamous cell carcinoma became a fibroblastic phenotype (EMT) and increased metastasis *in vivo* in the experimental metastasis model by injection into the tail vein of the mouse, even after several passages at pH*_e_* 7.4 (13). Adaptation to acidic pH*_e_* also altered fatty acid metabolism through sensitivity to PPARα (9).

Imaging technology has shown that the degree of the acidic pH*_e_* in tumors is not uniform throughout the tissue (14). The tumor cells face the different pH degree and respond differently for the degree such as mild (~pH*_e_* 6.8) and severe acidosis (<pH*_e_* 6.5). The study considered in this regard has been limited (11, 15, 16).

In this study, we performed cDNA microarray analysis of mouse B16-BL6 melanoma, which is resistant for the wide range of pH*_e_* degrees (4, 15, 17); the expression pattern of the genes induced/reduced by acidic pH*_e_* did not match the human metastatic melanoma signature, and more broad genes were affected. Through bioinformatics analysis, we found that the acidic pH*_e_* dependency of the tumors can be evaluated based on the correlation between acidic pH*_e_*-modulated gene expression of mouse B16-BL6 cells and patient prognosis. Details are given in the text.

## Materials and methods

### Cells and culture

Mouse B16-BL6 cells were kindly gifted from Dr. Kaoru Miyazaki (Yokohama City University, Japan) (17). Human cell lines consisting of melanoma (A2058 and A375C5), head and neck squamous cell carcinoma (HSC3, HSC4, and SAS), and lung cancer (A549, H1299, and HT1080) were obtained from the Japanese Collection of Research Bioresources Cell Bank (Osaka, Japan). They were cultured in a 1-to-1 mixture of Dulbecco’s modified Eagle’s medium (DMEM) (Life Technologies, Grand Island, NY, USA) and Ham’s F12 medium (Life Technologies) supplemented with 15 mM 4-(2-hydroxyethyl)-1- piperazine-ethanesulfonic acid (HEPES, pH*_e_* 7.4), 4 mM H_3_PO_4_ 1.8 g/L NaHCO_3_, 100 units/mL penicillin G (Meiji, Tokyo, Japan), 0.1 mg/mL streptomycin sulfate (Meiji, Tokyo, Japan), and 10% fetal bovine serum (HyClone, South Logan, UT, USA) in a humidified atmosphere in a 5% CO_2_ incubator. The pH*_e_* of the medium was adjusted to pH*_e_* 7.4 and 6.8 with NaOH and to pH*_e_* 5.9 with HCl for B16-BL6 cells (17). For human cell lines, pH*_e_* 7.4, 6.8, 6.5, and 6.2 media were used. Cell viability at each pH*_e_* was determined using the cell counting kit-8 (CCK-8, Dojindo Laboratories, Kumamoto, Japan) according to the manufacturer’s protocol.

### Data acquisition and analysis from public databases

The data sets of GSE52031 (18) and GSE8401 (19) were downloaded from the Gene Expression Omnibus (GEO) database (https://www.ncbi.nlm.nih.gov/geo/). The volcano plots were analyzed using the web-based tool VolcaNoseR (https://huygens.science.uva.nl/VolcaNoseR2/) (20). Gene Ontology (GO) enrichment analysis was performed using the web-based online software the Gene Ontology Resource Powered by PANTHER (https://geneontology.org/) (21). Gene expression in each pathological stage was determined using web-based software GEPIA2 (http://gepia2.cancer-pku.cn/#index) (22), and the prognostic signature of proteins was searched using the Human Protein Atlas database (http://www.proteinatlas.org).

### Acidic pH*_e_* treatment, RNA extraction, and cDNA microarray analysis

Confluent B16-BL6 cells were washed with Ca^2+^- and Mg^2+^-free phosphate-buffered saline (PBS(-)) and preincubated with serum-free DMEM/F12 (pH*_e_* 7.4) for overnight. They were treated with serum-free DMEM/F12 at pH*_e_* 7.4 as a control, pH*_e_* 6.8 and pH*_e_* 5.9 for B16-BL6 cells for 24 h (17). Total RNA from quadruplicate cultures was extracted with Isogen (Nippon gene, Tokyo, Japan) and subjected to the cDNA microarray analysis (4). A whole mouse genome microarray 4 × 44K (Agilent Technologies Inc., Santa Clara, CA, USA) was used, and cDNA microarray analysis using the two-color method (pH*_e_* 7.4 sample as the control was labeled with Cy3 (cyanine 3) and acidic pH*_e_* (6.8 or 5.9)-treated samples were labeled with Cy5) was performed by DNA Chip Research Inc. (Tokyo, Japan). The acidic pH*_e_*-modulated genes were selected by a fold change difference of 2 or more when up- or downregulated against pH*_e_* 7.4.

Human tumor cell lines that reached confluence were pretreated as described above, further treating cells with pH*_e_* 7.4 as a control, pH*_e_* 6.8, pH*_e_* 6.5, and pH*_e_* 6.2 for 24 h. Total RNA from triplicate cultures was extracted with Isogen and subjected to the reverse transcription-quantitative polymerase chain reaction (RT-qPCR), as described below.

### RT-qPCR

Total RNA was extracted with Isogen and reverse-transcribed to cDNA using a High-Capacity cDNA Reverse Transcription Kit (Thermo Fisher Scientific, Waltham, MA, USA). Target genes were amplified by GoTaq^®^ qPCR and RT-qPCR Systems (Promega, Madison, WI, USA) using the specific primers listed in **Supplementary Table S1**. The level of expression of each target gene was normalized relative to the level of *ACTB* mRNA in the same samples.

### Assessment of tumor acidic pH*_e_* dependency

We focused on the top 100 genes induced or reduced at acidic pH*_e_* (pH*_e_* 6.8 and pH*_e_* 5.9), respectively. If the acidic pH*_e_*-induced and -inhibited genes were correlated with shortened and prolonged survival time, respectively, we defined it as “hit” and counted the sum of hits of eight types of tumors, including the reversal case within 20%. The expression status of genes with a hit rate of 50% or more was also determined for correlation with staging.

### Statistical analysis

Simple comparison of the mRNA expression between two groups [pH*_e_* 7.4 versus acid pH*_e_* (6.8 or 5.9)] in the cDNA microarray analysis was determined by Student’s *t*-test. Further statistical significance of the web-based analysis was provided by the output. Significance of multiple comparisons was determined by Student’s *t*-test with Bonferroni’s multiple significance test correction and further confirmed by one-way analysis of variance (ANOVA) with *post hoc* Tukey’s honestly significant difference (HSD) test (https://astatsa.com/OneWay_Anova_with_TukeyHSD/). A 2 × 2 contingency was determined by chi-squared test. A p-value less than 0.05 was considered statistically significant. A p-value of less than 0.05 was considered statistically significant.

## Results

### Gene signature of acidic pH*_e_*

**Figure 1:**
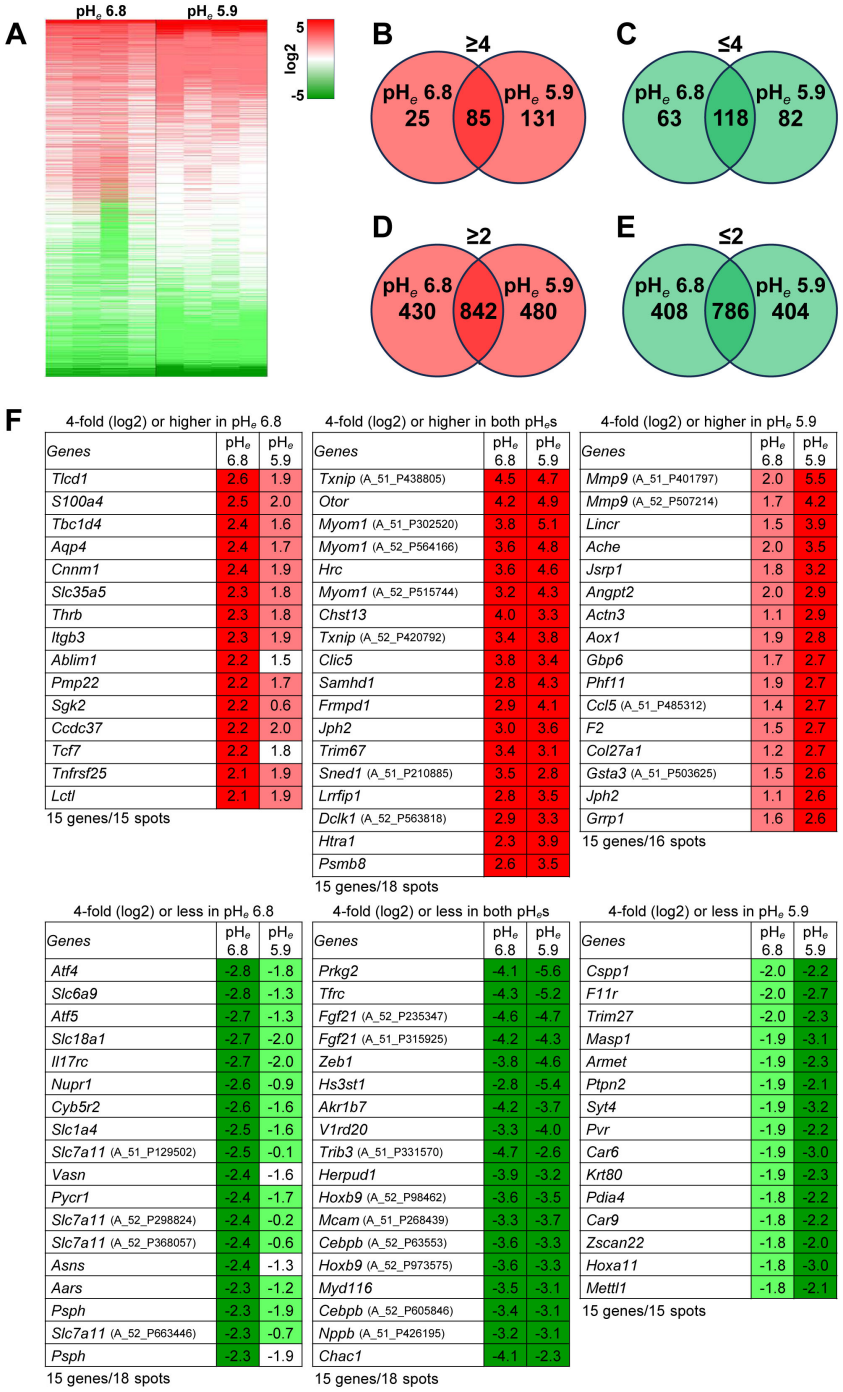
Acidic pH*_e_* modulates gene expression. **(A)** Heat map; number of genes expressed: ≥4-fold **(B)**, ≤4-fold **(C)**, ≥2-fold **(D)**, or ≤2-fold **(E)** (n=4). **(F)** The top 15 genes from each panel **(B, C)** were listed. The cDNA microarray data (GSE276124) is available on September 9, 2024.

**Figure 2:**
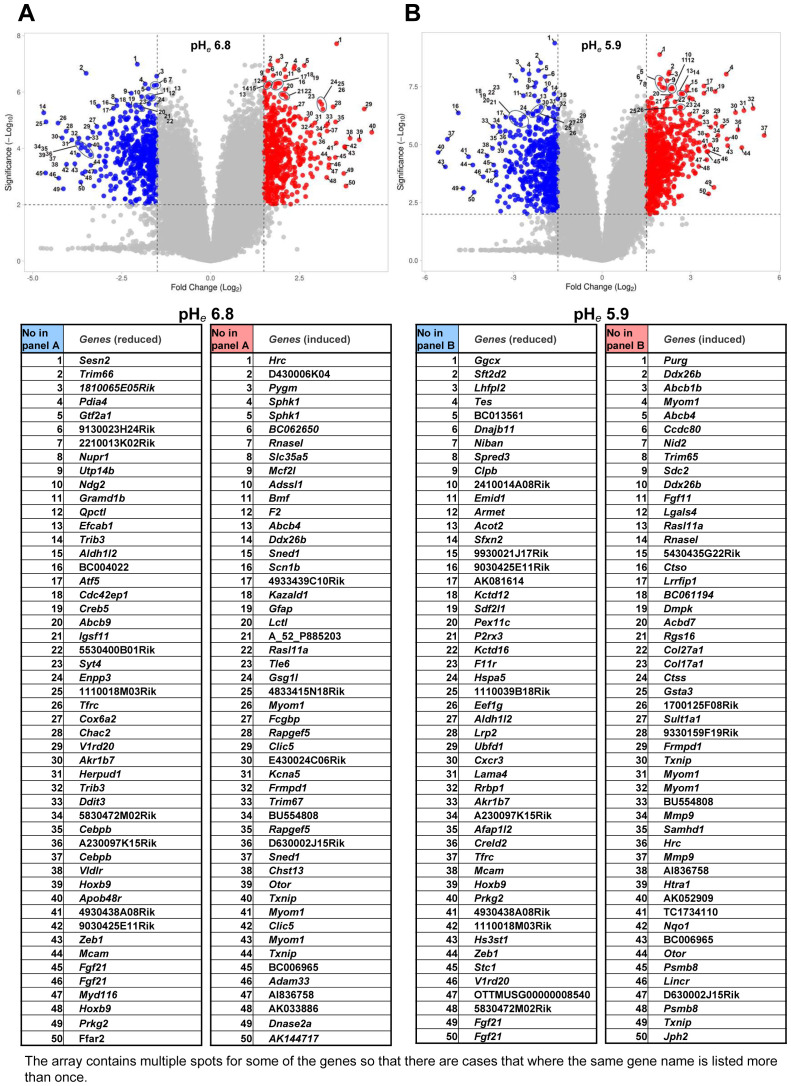
Volcano plots of acidic pH*_e_*-modulated gene expression. The 50 genes were numbered in the volcano plots are compatible with the following lists. **(A)** pH*_e_* 6.8 versus pH*_e_* 7.4; **(B)** pH*_e_* 5.9 versus pH*_e_* 7.4.

As shown previously, mouse B16 cells are extremely resistant to acidic pH*_e_* (**Supplementary Figure S1**) (4, 15, 17). We focused on the gene expression signature of acidic pH*_e_*-treated B16-BL6 cells at two different pH*_e_* levels, such as pH*_e_* 6.8 as mild acidosis and pH*_e_* 5.9 (optimal pH for matrix metalloproteinase 9 (type IV collagenase, gelatinase B, MMP9) induction (15) contributing to tumor metastasis) as severe acidosis. The heat map visualized that the gene expression affected many genes, that some genes have the same signature between pH*_e_* 6.8 and 5.9, and that some other genes are independently regulated (**Figure 1A**). The number of genes upregulated fourfold or more only at pH*_e_* 6.8 and 5.9 only for 25 and 131 genes, respectively, and the number of induced genes in both pH*_e_* s together was 85 genes (**Figure 1B**). The number of genes downregulated fourfold or less at pH*_e_* 6.8 and 5.9 alone for 63 and 82 genes, respectively, and the number of genes downregulated in both pH*_e_* s together was 118 genes (**Figure 1C**). When the numbers were counted in the case of twofold or higher, there were 430 and 480 genes at pH*_e_* 6.8 and 5.9 alone, respectively, and 842 genes common in both (**Figure 1D**); for twofold or less, there were 408 and 404 genes at pH*_e_* 6.8 and 5.9 alone, respectively, and 786 genes were common to both (**Figure 1E**). The representative top five genes are as follows: ≥4 at pH*_e_* 6.8 only, *Tlcd1*, *S100a4*, *Tbc1d4*, *Aqp4*, *Cnnm1*; ≥4 at both pH*_e_* s: *Txnip*, *Otor*, *Myom1*, *Hrc*, *Chst13*; ≥4 at pH*_e_* 5.9 only: *Mmp9*, *Lincr*, *Ache*, *Jsrp1*, *Angpt2*; ≤4 at pH*_e_* 6.8 only: *Atf4*, *Slc6a9*, *Atf5*, *Slc18a1*, *Il17rc*; ≤4 at both pH*_e_* s: *Prkg2*, *Tfrc*, *Fgf21*, *Zeb1*, *Hs3st1*; ≤4 at pH*_e_* 5.9 only: *Cspp1*, *F11r*, *Trim27*, *Masp1*, *Armet* (**Figure 1F**). The volcano plot showed that two degrees of acidic pH*_e_* significantly affected mRNA expression and the difference of acidic pH*_e_* degree independently affected some gene expressions (**Figure 2**).

### Gene ontology analysis

**Figure 3:**
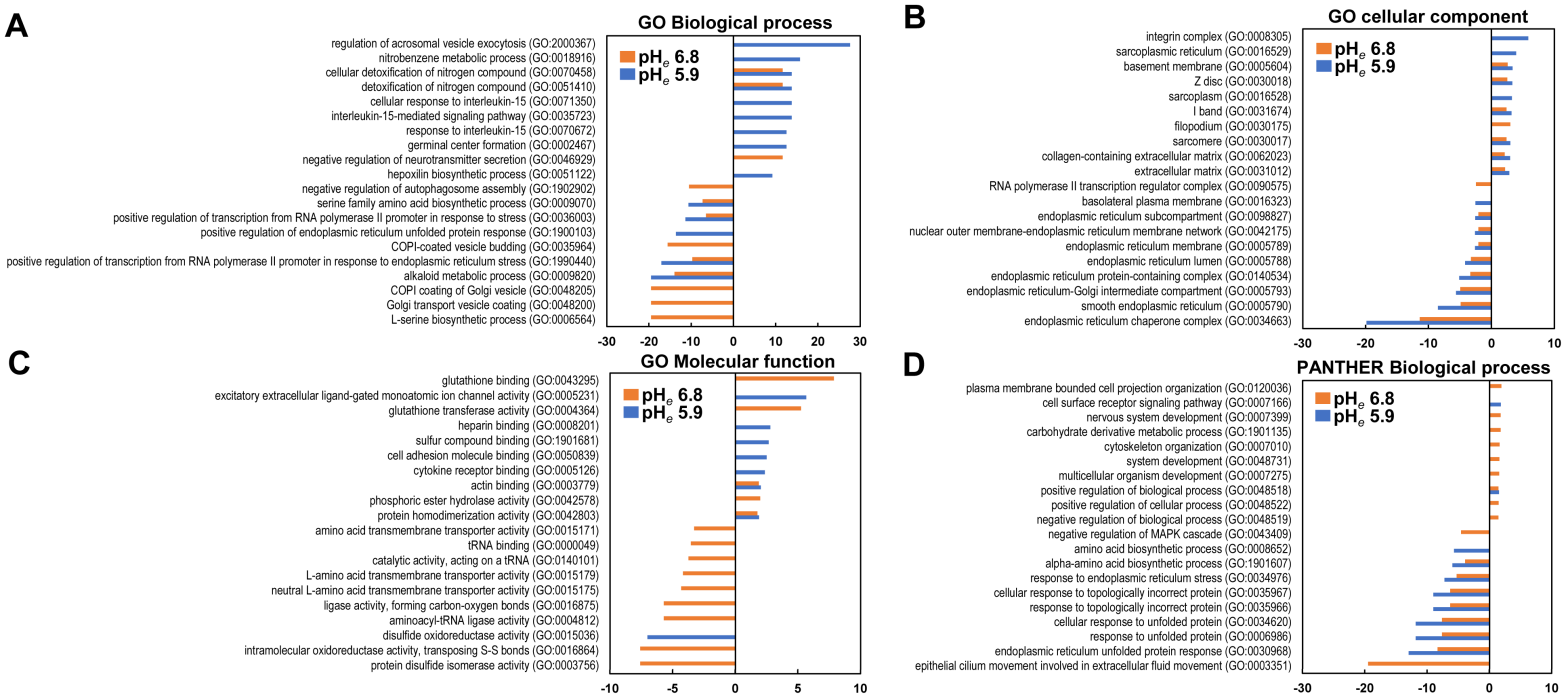
Gene ontology analysis. The top items are listed in each category as up- or downregulated genes. **(A)** GO Biological Process; **(B)** Cellular Components; **(C)** GO Molecular Function; **(D)** PANTHER Molecular Function. The enrichment of induced and reduced genes is shown on the plus and minus axis, respectively.

Gene ontology analysis revealed the effect of mild and severe acidosis, which independently and jointly affected the cells (**Figure 3**, **Supplementary Tables S2**, **S3**). In the GO biological process, the genes induced by pH*_e_* 5.9 were mainly enriched in GO:2000367 (regulation of acrosomal vesicle exocytosis) and GO:0018916 (nitrobenzene metabolic process), followed by GO:0070458 (cellular detoxification of nitrogen compound) and GO:0051410 (detoxification of nitrogen compound) (**Figure 3A**). In the latter two categories, the genes induced by pH*_e_* 6.8 were also commonly enriched. The enrichment in GO:0046929 (negative regulation of neurotransmitter secretion) was only observed at pH*_e_* 6.8. On the contrary, the genes reduced at pH*_e_* 6.8 were enriched in GO:0006564 (L-serine biosynthetic process), GO:0048200 (Golgi transport vesicle coating), GO:0048205 (COPI (a coatomer, a protein complex) coating of Golgi vesicle), followed by GO:0009820 (alkaloid metabolic process) and GO:1990440 (positive regulation of transcription from the RNA polymerase II promoter in response to endoplasmic reticulum stress). Enrichment of the latter two categories was also observed at pH*_e_* 5.9. In the GO cellular component section, the genes induced by pH*_e_* 5.9 were only frequently enriched in GO:0008305 (integrin complex) and GO:0016529 (sarcoplasmic reticulum) and commonly enriched with pH*_e_* 6.8 in GO:0005604 (basement membrane) and GO:0030018 (Z disc) (**Figure 3B**). Integrin activity plays an important role in the metastatic process. Therefore, it is reasonable to understand that acidic pH affects metastatic behavior. The genes reduced by both pH*_e_* s were commonly enriched in GO:0034663 (endoplasmic reticulum chaperone complex) and GO:0005790 (smooth endoplasmic reticulum). In the GO molecular function section, the genes induced by pH*_e_* 6.8 and pH*_e_* 5.9 were independently enriched in GO:0043295 (glutathione binding) and GO:0005231 (excitatory extracellular ligand-gated monoatomic ion channel activity) respectively (**Figure 3C**). The genes that were reduced at pH*_e_* 5.9 were only enriched in GO:0015036 (disulfide oxidoreductase activity). Interestingly, the genes reduced by pH*_e_* 6.8 were broadly enriched: *e.g.*, GO:0003756 (protein disulfide isomerase activity) and GO:0016864 (intramolecular oxidoreductase activity, transposing S-S bonds). Overall, the induced genes were enriched at pH*_e_* 5.9 and the reduced genes were enriched at pH*_e_* 6.8.

In the PANTHER ontology, there was no enrichment of more than two enrichment values in the induced gene in one or both conditions in the biological process analysis (**Figure 3D**). The enrichment score was low but broad in the other ontology category (**Supplementary Tables S2**, **S3**). Thus, acidic pH*_e_* also affects gene expression and contributes to a wide range of cellular functions.

### Comparison of acidic pH*_e_*-modulated genes with other mouse models

We then compared with the modulation of gene expression by acidic pH*_e_* with the genes in the spontaneous mouse melanoma established by a tamoxifen-driven B-RAF/PTEN (18). High-expression genes in metastatic tumor cells (Mets) were induced by either pH*_e_* 6.8 or pH*_e_* 7.4, but low-expression genes in Mets were not often modulated by acidic pH*_e_* s. In the circulating tumor cells (CTC), the majority of both acidic pH*_e_*-induced genes were distributed in low-expression genes in CTC versus primary, that is, inversely correlated; these similarly tended to be with the reduced genes in CTC less than Mets (**Table 1**).

**Table 1:** Acidic pH*_e_*-modulated genes compared with the spontaneous mouse melanoma established by a tamoxifen-driven B-RAF/PTEN (18).

Gene expression status of GSE8401 data set		pH*_e_* 7.4 vs. pH*_e_* 6.8			pH*_e_* 7.4 vs. pH*_e_* 5.9		
		≥2-fold	≤2-fold	*P* value	≥2-fold	≤2-fold	*P* value
Mets *versus* primary	High expression (2193 genes)	119	68	} <0.05	71	44	} <0.05
	Low expression (333 genes)	14	10		11	8	
CTC *versus* primary	High expression (1011 genes)	71	43	} <0.05	47	30	} <0.05
	Low expression (10541 genes)	822	334		533	233	
CTC *versus* Mets	High expression (649 genes)	53	31	} <0.05	40	26	} <0.05
	Low expression (12203 genes)	928	377		592	256	

Mets, metastasized tumor cells; CTC, circulating tumor cell.

### Acidic pH*_e_* signature and the other mouse and human melanomas

**Figure 4:**
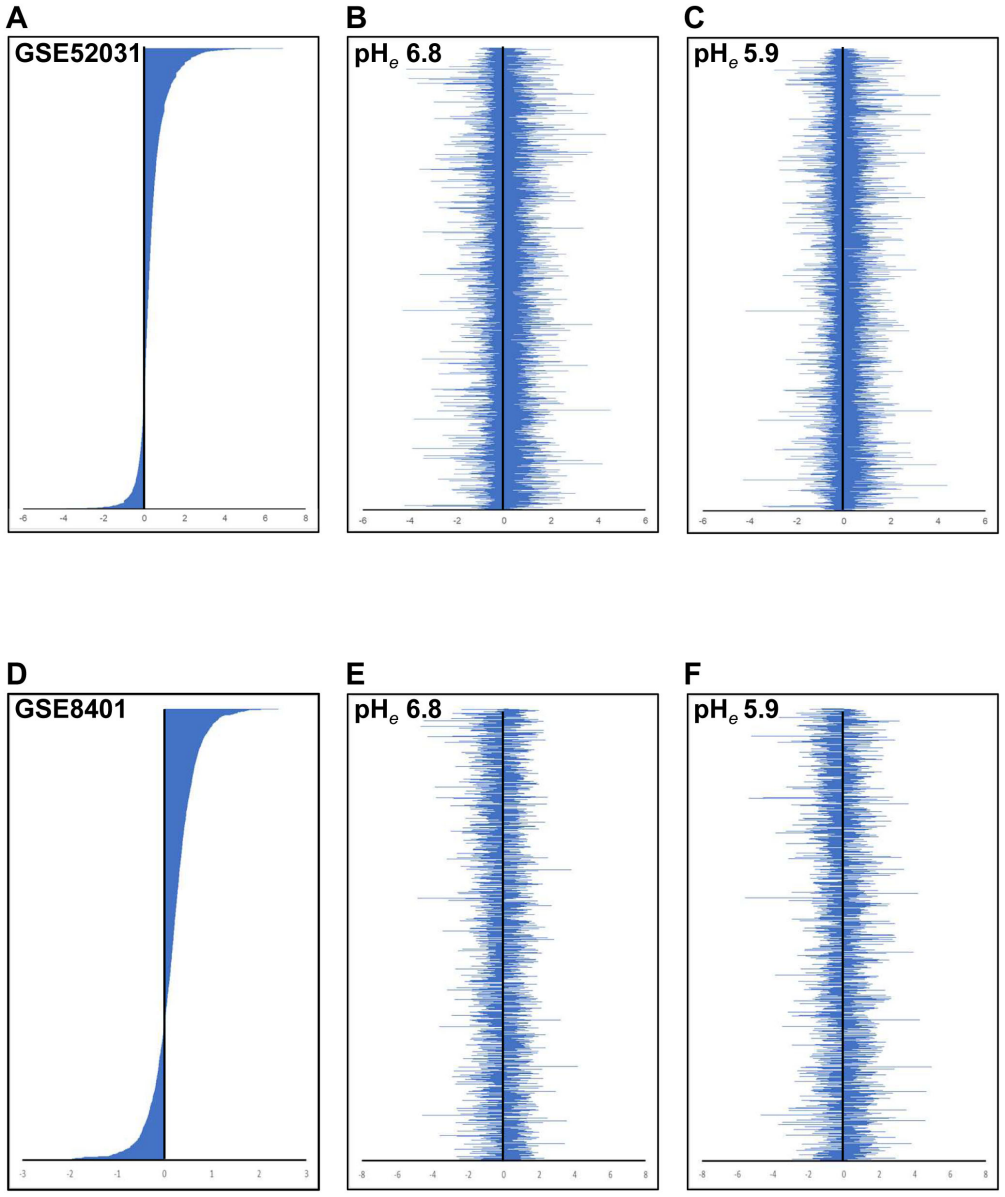
Comparison of expression signature between public data and acidic pH*_e_*-altered gene expression. The gene expression signature of GSE52031 **(A)** and GSE8401 **(B)** is shown in descending order. The acidic pH*_e_*-modulated gene expression signature was shown [**(B, E)** for pH*_e_* 6.8; **(C, F)** for pH*_e_* 5.9]. The order of genes in **(B, C)** was listed as the same as **(A)**, and in **(E, F)** as the same as **(D)**.

We compared with the distribution of gene expression modulated in Mets. Unexpectedly, the distribution of gene expression at both acidic pH*_e_* s was not correlated with melanomas in spontaneous mouse models (**Figures 4A–C**) and human clinical samples (**Figures 4D–F**). Because the modulation of gene expression was seen throughout, we speculated that acidic pH*_e_*-modulated genes that appeared in this study are common throughout the tumor origin.

### Limitation of the correlation between the acidic pH*_e_*-induced genes and pathological staging of the patients

**Figure 5:**
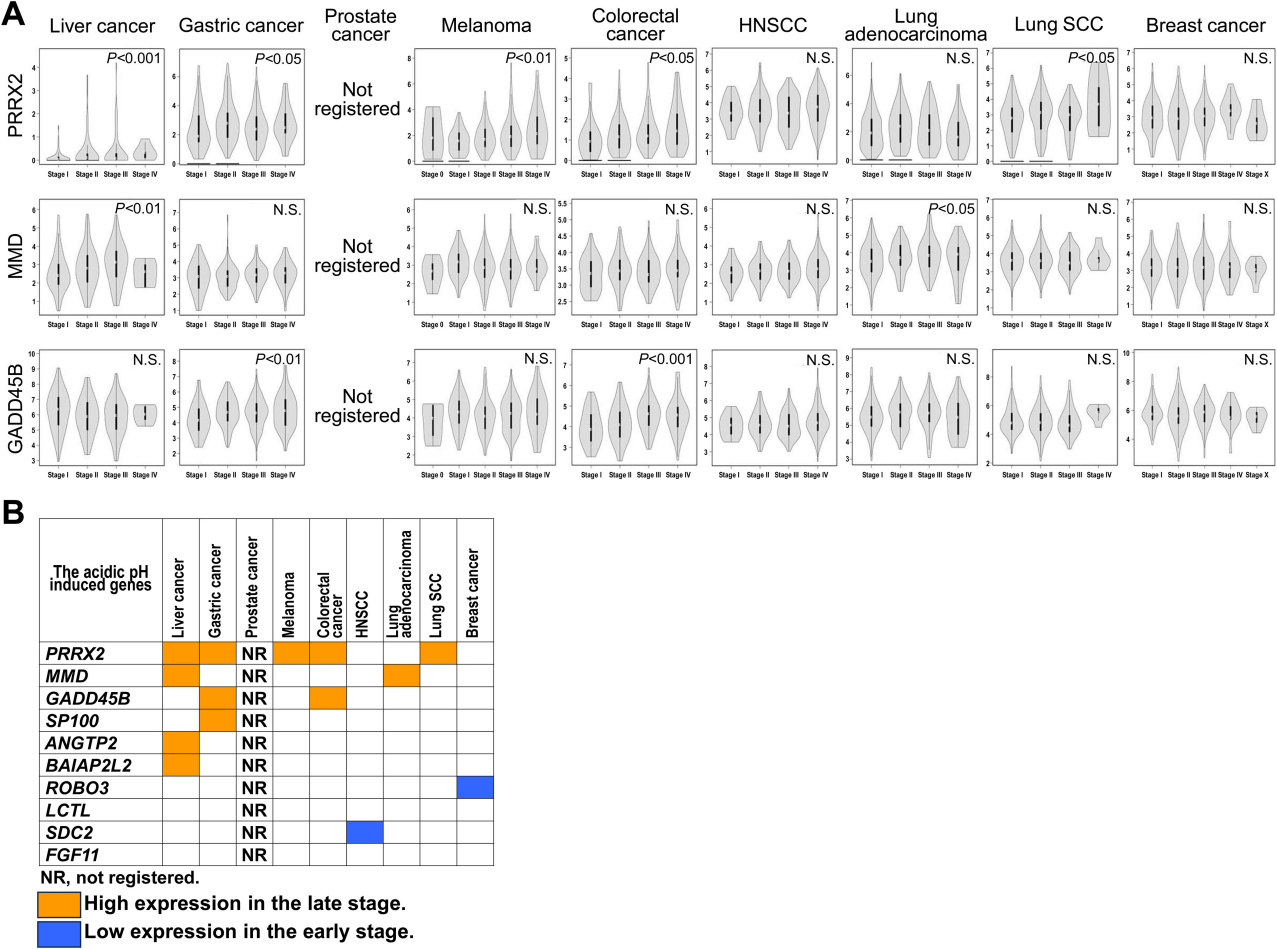
Correlation of acidic pH*_e_* modulating genes with pathological stage of the patients. **(A)** The violin graph of pathological stages in the three representative genes. **(B)** Summary of the correlation.

To confirm the role of acidic pH*_e_*-induced genes in the tumor progression, we evaluated whether the acidic pH*_e_*-induced genes were correlated with pathological staging. **Figure 5** shows that *PRRX2* gene expression was high in the late stage of 4/7 tumors followed by *MMD* and *GADD45B* (2/7 tumors). Thus, the correlation of the acidic pH*_e_*-induced gene expression with pathological stages in seven tumors is limited, and overall, the majority of the acidic pH*_e_*-induced gene expression was not correlated with the pathological staging of the patients.

### Acidic pH*_e_* dependency of eight types of tumors

**Figure 6:**
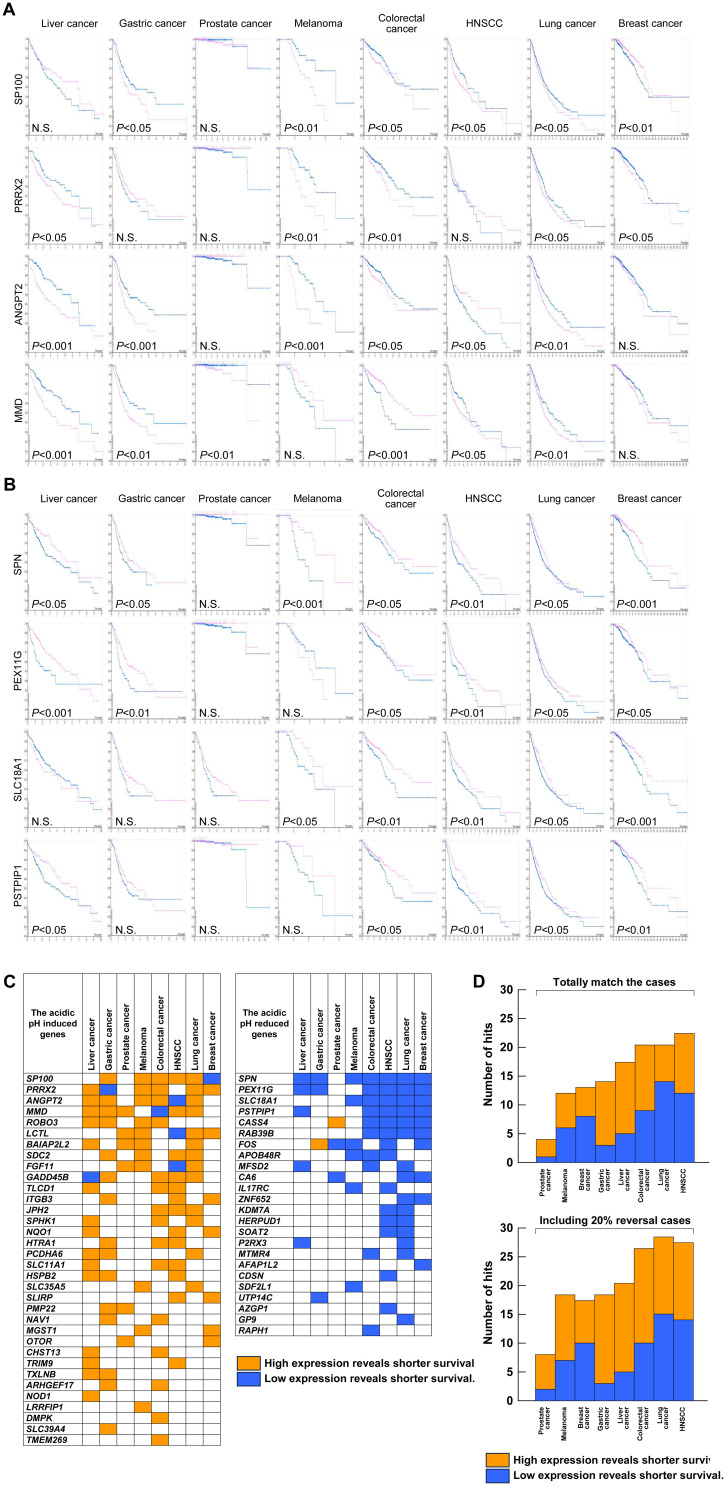
Prognostic significance of the acidic pH*_e_*-modulated genes. The Kaplan–Meier plot of four representative genes: **(A)** acidic pH*_e_*-induced genes; **(B)** acidic pH*_e_*-reduced genes. **(C)** Distribution list of “hit” genes. **(D)** Summary of the total number of hits excluding (*upper*) and including an inversion case of up to 20% (*lower*) from **(C)**.

Next, we focused on 100 genes that were modulated by acidic pH*_e_* to correlate with the patent survival using the Protein Atlas database. **Figure 6** shows the five representative genes each in the induced (A) and reduced (B) by the acidic pH*_e_* in eight kinds of human tumors including melanoma. In **Figures 6C, D**, the summarized data represent the acidic pH*_e_*-induced genes showing shorter survival and the acidic pH*_e_*-reduced showing longer survival, which were defined as the “hit”. Interestingly, among eight neoplasms, the hit number of melanoma did not have the highest frequency. The hit numbers were lowest in prostate cancer and highest in colorectal cancer, lung cancer, and HNSCC. The acidic pH*_e_*-induced genes are dominant in gastric and liver cancers, and the acidic pH*_e_*-reduced genes are rather dominant in the breast cancer. Thus, both gastric and liver cancers could be categorized as the acidic pH*_e_*-induced type and breast cancer as the acidic pH*_e_*-reduced type. Finally, we validated that the acidic pH-responsive signature of B16-BL6 is suitable for evaluating the tumor acidic pH dependency for prognosis; several tumor cell lines are tested for the acidic pH response (**Supplementary Figure S2**). All cell lines respond to acidic pH, but the positive or negative responses are different. At least in the present study, the acidic pH*_e_*-modulated gene list of B16-BL6 cells was suggested to be useful for evaluating the acidic pH*_e_* dependency. This is the first report that provides new insights into the assessment of acidic pH dependency of tumors.

## Discussion

Acidic pH*_e_* is known to promote malignant tumor phenotype such as EMT, invasion, and metastasis (12, 13, 23, 24). The acidic pH*_e_* sensing system of the cells such as proton sensing GPCRs such as GPR4, TDAG8, and OGR1 is a good strategy for tumor therapy (25). Majority of their functions are tumor suppressors (26) so their inhibition can cause good prognosis. We have identified transient receptor M5 (TRPM5) involved in acidic pH*_e_* sensing, and the pharmacological treatment of the implanted B16-BL6 cells in the mouse with the inhibitor reduced lung metastasis in the B16-BL6 model (17). Further studies are needed for clinical trials.

Another established strategy is alkalinization therapy. Administration of NaHCO_3_ resulted in a more alkaline pH*_e_* in the tumor tissues than normal tissues due to decreasing buffering effect (2, 3). In a mouse xenograft model with the metastatic breast cancer cell line MDA-MB-231, oral administration of NaHCO_3_ inhibited metastasis and survival of the mice (27). Acidic pH*_e_* increased a level of the immune checkpoint molecule programmed cell death protein 1 (PD-L1), and administration of NaHCO_3_ in allogeneic transplanted mice reduced tumor growth, suggesting escape from the immune recognition (28). Thus, the mouse model has reported readiness leading to the clinical use of NaHCO_3_ for tumor therapy. In fact, the phase 1 clinical trials (NCT01350583, NCT01198821, and NCT01846429) were registered in the USA (http://www.clinicaltrials.gov) and reported as follows: among them, the study (NCT01846429) showed that administration of NaHCO_3_ reduced the perceived pain level by approximately 30% within the first 3 weeks, and the reduction was maintained when therapy was continued for more than 6 weeks (29). As an alternative to buffer therapy, the use of free base lysine has been reported by Bailey et al. (30). They also investigated a potential mechanism underlying the efficacy of buffer therapy (31). Specifically, when using free base lysine, buffer therapy shows efficacy in reducing the metastatic ability of acid-producing cells whose metastatic phenotype is supported by the formation of an acidic microenvironment. However, this therapy is not effective for cells that constitutively produce proteinases, such as matrix metalloproteinases, that contribute to metastasis.

Alkalinization affects efficacy of the chemotherapeutic agents. For example, the efficacy of the weak base drugs was decreased by acidic pH*_e_* and they are expected to be increased by alkalization. Raghunand et al. (32) demonstrated that alkalization therapy with NaHCO_3_ increased the efficacy of weak base drugs such as doxorubicin in a mouse model. Hamaguchi et al. (33) successfully demonstrated that the combination regimen consisting of oxaliplatin, irinotecan, fluorouracil, and leucovorin (FOLFIRINOX) with administration of an alkaline diet and NaHCO_3_ successfully increased in the pH*_e_*, which were monitored in the urine, and prolonged the survival of the patients with pancreatic cancer as compared to FOLFIRINOX alone.

In this study, we investigated how acidic pH*_e_* affects cellular functions that are independently and commonly regulated by the different degrees of acidosis. Since pH*_e_* in tumor tissue is not uniform (14), it was necessary to determine which of the cells and which of the molecules to target for therapeutic strategy. Furthermore, acidic pH*_e_* dependency of tumors was shown for the first time using the Human Protein Atlas based on the microarray analysis in this study. Acidic pH*_e_* effect does not enhance cell type-specific gene regulation; more generally, however, acidic pH*_e_* supports metastatic phenotypes observed in a previous report (17). Although we expected a good correlation of acidic pH*_e_*-modulated gene signature with that of human melanoma, colorectal and lung cancer and HNSCC were more relevant than melanoma, suggesting that alkalinization therapy is highly recommended concomitantly with conventional chemotherapy. On the other hand, the number of hits is lowest in prostate cancer, suggesting that prostate cancer is difficult to optimize for alkalinization therapy compared to the other tumor types.

The acidic pH*_e_*-induced genes are mainly hit for gastric and liver cancer, but the opposite is true for breast cancer. For the clinical therapeutic strategy, overexpression by gene transfer, except DNA vaccine (34), can hardly be developed at present, but alkalization therapy combination with the conventional molecular specific inhibitor or antibody medicine is highly applicable. Especially for gastric and liver cancer, the combination of alkalinization therapy with drugs is expected to tend to the gene-inducible type due to the acidic pH*_e_* dependency. *SP100*, *PRRX2*, and *ANGPT2* are commonly correlated with poor prognosis among five out of eight tumors. Since the reduction of SP100 was reported to be induced by radioresistance of colorectal cancer (35), the combination of alkalinization therapy with radiation may provide a good prognosis. Also, upregulation of SP100 was found by ursodeoxycholic acid combined with prednisolone and immunosuppressive triple therapy (36), suggesting that it can be combined with alkalinization therapy. CircRNA is also a promising tool for future cancer therapy (37). For example, circLRFN5 is expected to be combined with alkalinization therapy because *Prrx2* expression was highly induced by acidic pH*_e_* in this study and PRRX2 had inhibitory activity of ferroptosis in glioblastoma (38).

In conclusion, the acidic pH*_e_* signature of B16-BL6 is not limited to melanoma but can be adapted to many tumors. Our results showed that acidic pH*_e_* contributes to poor survival of patients with a wide range of tumor types, and also that tumors can be classified by their response to acidic pH*_e_*. The tumor classification based on the response to acidic pH*_e_* will provide new insight into the strategy of chemotherapy and gene therapy for patients with tumors.

## Data Availability

The cDNA microarray data have been deposited at NCBI (GSE276124, https://www.ncbi.nlm.nih.gov/geo/query/acc.cgi?acc=GSE276124), which is available on Sept 9, 2024. Further inquiries can be directed to the corresponding author.

## Supplementary material

Supplementary Figure 1 Viability of B16-BL6 cells in serum-free medium with different pH*_e_*. Cells (1 × 10^4^ cells/well) were seeded into the 96-well culture plate. After confirming that the cells adhered and spread on the vessels (3 h after inoculation), the cells were incubated in a serum-free medium with different pH*_e_* s. At the end of the incubation, cells were treated with CCK-8 dye for 1.5 h. Absorbance was measured at 450 nm and plotted after subtracting the background absorbance without the cells at each pH*_e_*. ***P*<0.01.

Supplementary Figure 2 RT-qPCR for human tumor cell lines. Each human tumor cell line was grown to confluence in 10% FBS containing medium in two six-well plates. They were then preincubated overnight in serum-free medium at pH*_e_* 7.4 and stimulated at different acidic pH*_e_* s (6.8, 6.5, and 6.2) for 24 h. The pH*_e_* 7.4 medium was used as a control, and experiments were performed in triplicate. After incubation, total RNA was extracted, reverse transcribed, and subjected to qPCR analysis using specific primer sets. **P*<0.05; ***P*<0.01.
