# Supplementary figures and images for: Clinical significance of acidic extracellular microenvironment modulated genes
Source: Front Oncol. 2024 Sep 20;14:1380679. doi: 10.3389/fonc.2024.1380679 (PMC11449683; doi:10.3389/fonc.2024.1380679)

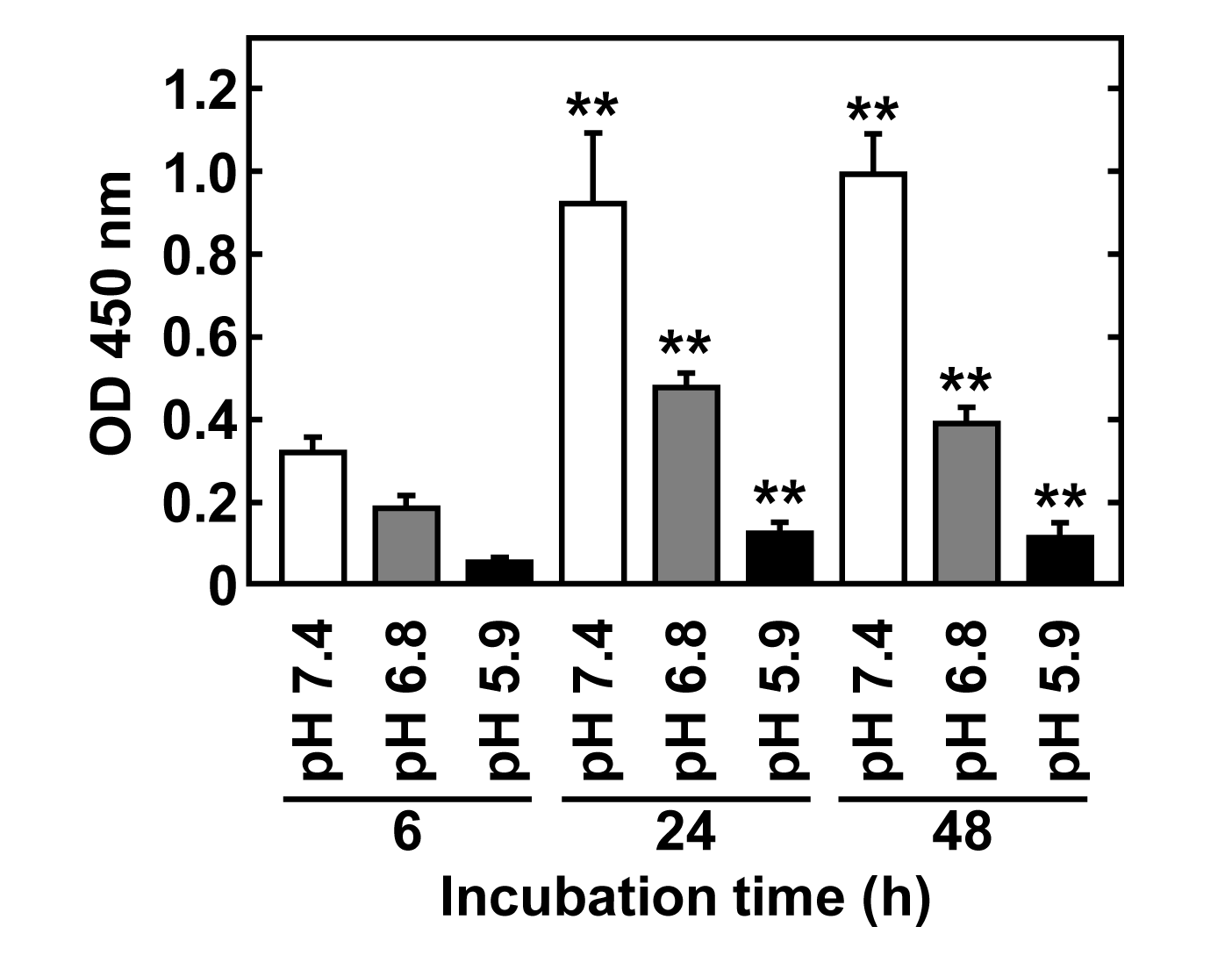

Supplement: Supplementary Figure 1 — Viability of B16-BL6 cells in serum-free medium with different pH e . Cells (1 × 10^4^ cells/well) were seeded into the 96-well culture plate. After confirming that the cells adhered and spread on the vessels (3 h after inoculation), the cells were incubated in a serum-free medium with different pH e s. At the end of the incubation, cells were treated with CCK-8 dye for 1.5 h. Absorbance was measured at 450 nm and plotted after subtracting the background absorbance without the cells at each pH e . **P<0.01. [file Image1.tif]

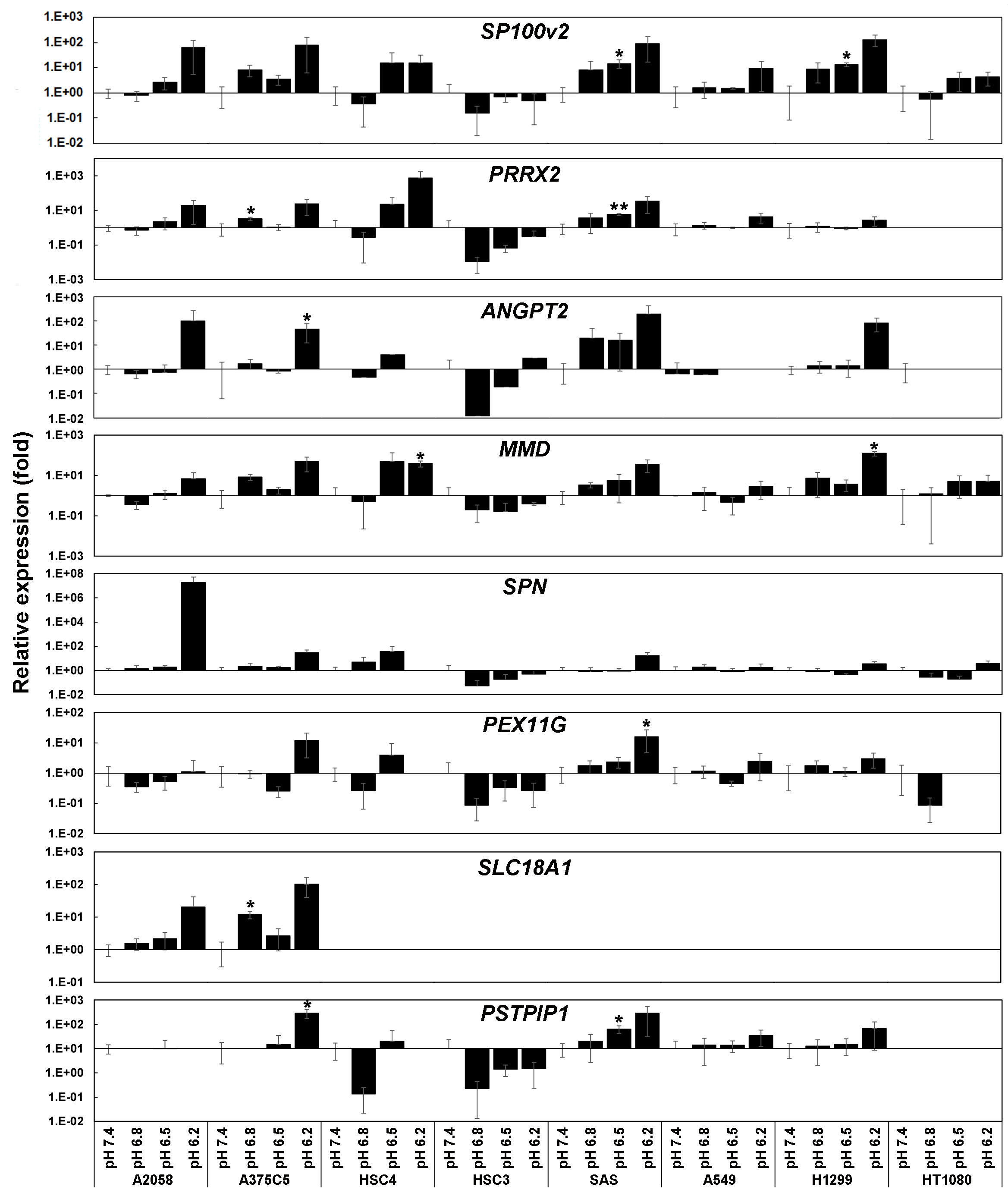

Supplement: Supplementary Figure 2 — RT-qPCR for human tumor cell lines. Each human tumor cell line was grown to confluence in 10% FBS containing medium in two six-well plates. They were then preincubated overnight in serum-free medium at pH e 7.4 and stimulated at different acidic pH e s (6.8, 6.5, and 6.2) for 24 h. The pH e 7.4 medium was used as a control, and experiments were performed in triplicate. After incubation, total RNA was extracted, reverse transcribed, and subjected to qPCR analysis using specific primer sets. *P<0.05; **P<0.01. [file Image2.tif]
