# Supplementary material for: Clinical significance of acidic extracellular microenvironment modulated genes
Source: Front Oncol. 2024 Sep 20;14:1380679. doi: 10.3389/fonc.2024.1380679 (PMC11449683; doi:10.3389/fonc.2024.1380679)
Supplement: Supplementary file 3 [file Table1.docx]

Table S1. Primer sequences for RT-qPCR

| Target genes Sequences |
| --- |
| *SP100v2* Forward: 5'-AGATATGGCAAAGGCGGACA-3' |
| Reverse: 5'-CCTCTTGGGTGCATTGGGAT-3' |
|  |
| *PRRX2* Forward: 5'-GCCGCAGGATGGTGAGTG-3' |
| Reverse: 5'-GTTGGCTGCTGTTGAACGTG-3' |
|  |
| *ANGPT2* Forward: 5'-GATGTCCACATCAAACTCTAAGGAC-3' |
| Reverse: 5'-GTTAACGTGTAGATGCCATTCGT-3' |
|  |
| *MMD* Forward: 5'-AAGGACAGTGGAGCATTGTTTTC-3' |
| Reverse: 5'-TTCACGAAGATTTAACCATGGAGC-3' |
|  |
| *SPN* Forward: 5'-GTCTTGCCCCAGCCTCG-3' |
| Reverse: 5'-GCTGGGACCGGCTCG-3' |
|  |
| *PEX11G* Forward: 5'-GGGACCGCCTGATCCGA-3' |
| Reverse: 5'-CAACAGACGTGTCCCCACTT-3' |
|  |
| *SLC18A1* Forward: 5'-ACATGGGCTCCAAGTGAGTT-3' |
| Reverse: 5'-TCCTGTGACAGCTACAGGTCT-3' |
|  |
| *PSTPIP1* Forward: 5'-TGCGGCATGATAAAGAGGTTCT-3' |
| Reverse: 5'-CAGAAGCTGCCAACGAAGTG-3' |
|  |
| *ACTB* Forward: 5'-TGGCACCCAGCACAATGAA-3' |
| Reverse: 5'-CTAAGTCATAGTCCGCCTAGAAGCA-3' |
