# Supplementary material for: Clinical significance of acidic extracellular microenvironment modulated genes
Source: Front Oncol. 2024 Sep 20;14:1380679. doi: 10.3389/fonc.2024.1380679 (PMC11449683; doi:10.3389/fonc.2024.1380679)
Supplement: Supplementary file 4 [file Table2.docx]

| **Table S2. GO analysis.** | | | | | | | | | | | | |
| --- | --- | --- | --- | --- | --- | --- | --- | --- | --- | --- | --- | --- |
|  |  | **Category** | **Fold  enrichment** | **P-value** | **FDR*** |  |  |  | **Category** | **Fold  enrichment** | **P-value** | **FDR*** |
| **Up-regulation at pH*_e_* 6.8** | | |  |  |  | **Down-regulation at pH*_e_* 6.8** | | | |  |  |  |
|  | ***GO biological process complete*** | |  |  |  |  |  | ***GO biological process complete*** | |  |  |  |
|  |  | detoxification of nitrogen compound (GO:0051410) | 11.69 | 3.1E-04 | 2.0E-02 |  |  |  | L-serine biosynthetic process (GO:0006564) | 19.51 | 3.4E-04 | 1.8E-02 |
|  |  | cellular detoxification of nitrogen compound (GO:0070458) | 11.69 | 3.1E-04 | 1.9E-02 |  |  |  | COPI coating of Golgi vesicle (GO:0048205) | 19.51 | 3.4E-04 | 1.7E-02 |
|  |  | negative regulation of neurotransmitter secretion (GO:0046929) | 11.69 | 3.1E-04 | 1.9E-02 |  |  |  | Golgi transport vesicle coating (GO:0048200) | 19.51 | 3.4E-04 | 1.7E-02 |
|  |  | neuronal action potential propagation (GO:0019227) | 8.50 | 9.1E-04 | 4.8E-02 |  |  |  | COPI-coated vesicle budding (GO:0035964) | 15.60 | 5.8E-04 | 2.8E-02 |
|  |  | mitotic DNA replication checkpoint signaling (GO:0033314) | 8.50 | 9.1E-04 | 4.8E-02 |  |  |  | alkaloid metabolic process (GO:0009820) | 13.93 | 1.6E-04 | 9.9E-03 |
|  |  | action potential propagation (GO:0098870) | 8.50 | 9.1E-04 | 4.8E-02 |  |  |  | negative regulation of autophagosome assembly (GO:1902902) | 10.50 | 2.9E-05 | 2.5E-03 |
|  |  | middle ear morphogenesis (GO:0042474) | 5.76 | 2.4E-04 | 1.7E-02 |  |  |  | positive regulation of transcription from RNA polymerase II promoter in response to endoplasmic reticulum stress (GO:1990440) | 9.75 | 1.5E-04 | 9.3E-03 |
|  |  | secondary alcohol biosynthetic process (GO:1902653) | 5.02 | 4.9E-05 | 4.8E-03 |  |  |  | proline transport (GO:0015824) | 8.13 | 1.0E-03 | 4.3E-02 |
|  |  | cholesterol biosynthetic process (GO:0006695) | 5.02 | 4.9E-05 | 4.8E-03 |  |  |  | chondrocyte proliferation (GO:0035988) | 8.03 | 1.1E-04 | 7.0E-03 |
|  |  | basement membrane organization (GO:0071711) | 4.95 | 2.6E-04 | 1.8E-02 |  |  |  | serine family amino acid biosynthetic process (GO:0009070) | 7.31 | 5.1E-04 | 2.4E-02 |
|  |  |  |  |  |  |  |  |  |  |  |  |  |
|  | ***GO molecular function complete*** | |  |  |  |  |  | ***GO molecular function complete*** | |  |  |  |
|  |  | glutathione binding (GO:0043295) | 7.88 | 4.0E-05 | 6.7E-03 |  |  |  | protein disulfide isomerase activity (GO:0003756) | 7.59 | 1.4E-04 | 1.7E-02 |
|  |  | glutathione transferase activity (GO:0004364) | 5.26 | 1.7E-04 | 2.3E-02 |  |  |  | intramolecular oxidoreductase activity, transposing S-S bonds (GO:0016864) | 7.59 | 1.4E-04 | 1.7E-02 |
|  |  | phosphoric ester hydrolase activity (GO:0042578) | 2.00 | 2.2E-04 | 2.7E-02 |  |  |  | ligase activity, forming carbon-oxygen bonds (GO:0016875) | 5.71 | 7.1E-06 | 1.5E-03 |
|  |  | actin binding (GO:0003779) | 1.88 | 1.6E-04 | 2.1E-02 |  |  |  | aminoacyl-tRNA ligase activity (GO:0004812) | 5.71 | 7.1E-06 | 1.4E-03 |
|  |  | calcium ion binding (GO:0005509) | 1.83 | 2.1E-05 | 4.0E-03 |  |  |  | neutral L-amino acid transmembrane transporter activity (GO:0015175) | 4.33 | 2.9E-04 | 2.9E-02 |
|  |  | protein homodimerization activity (GO:0042803) | 1.77 | 5.1E-06 | 1.4E-03 |  |  |  | L-amino acid transmembrane transporter activity (GO:0015179) | 4.18 | 1.4E-05 | 2.5E-03 |
|  |  | zinc ion binding (GO:0008270) | 1.70 | 8.6E-05 | 1.3E-02 |  |  |  | catalytic activity, acting on a tRNA (GO:0140101) | 3.75 | 1.3E-07 | 4.8E-05 |
|  |  | identical protein binding (GO:0042802) | 1.65 | 1.6E-12 | 1.6E-09 |  |  |  | tRNA binding (GO:0000049) | 3.55 | 1.3E-04 | 1.6E-02 |
|  |  | transition metal ion binding (GO:0046914) | 1.62 | 2.9E-05 | 5.2E-03 |  |  |  | amino acid transmembrane transporter activity (GO:0015171) | 3.29 | 1.6E-04 | 1.8E-02 |
|  |  | adenyl nucleotide binding (GO:0030554) | 1.59 | 4.5E-07 | 1.9E-04 |  |  |  | phosphoprotein phosphatase activity (GO:0004721) | 2.67 | 8.9E-05 | 1.2E-02 |
|  |  |  |  |  |  |  |  |  |  |  |  |  |
|  | ***GO cellular component complete*** | |  |  |  |  |  | ***GO cellular component complete*** | |  |  |  |
|  |  | filopodium (GO:0030175) | 3.02 | 2.3E-04 | 9.7E-03 |  |  |  | endoplasmic reticulum chaperone complex (GO:0034663) | 11.38 | 1.9E-05 | 1.3E-03 |
|  |  | lamellipodium (GO:0030027) | 2.72 | 1.6E-05 | 1.1E-03 |  |  |  | endoplasmic reticulum-Golgi intermediate compartment (GO:0005793) | 4.94 | 5.3E-08 | 7.1E-06 |
|  |  | basement membrane (GO:0005604) | 2.60 | 9.9E-04 | 3.6E-02 |  |  |  | smooth endoplasmic reticulum (GO:0005790) | 4.88 | 6.0E-04 | 3.0E-02 |
|  |  | contractile fiber (GO:0043292) | 2.55 | 4.8E-06 | 4.8E-04 |  |  |  | endoplasmic reticulum protein-containing complex (GO:0140534) | 3.35 | 2.4E-06 | 2.0E-04 |
|  |  | Z disc (GO:0030018) | 2.53 | 1.0E-03 | 3.7E-02 |  |  |  | endoplasmic reticulum lumen (GO:0005788) | 3.25 | 1.7E-04 | 9.6E-03 |
|  |  | I band (GO:0031674) | 2.42 | 9.6E-04 | 3.6E-02 |  |  |  | RNA polymerase II transcription regulator complex (GO:0090575) | 2.44 | 1.9E-05 | 1.3E-03 |
|  |  | sarcomere (GO:0030017) | 2.41 | 1.1E-04 | 5.1E-03 |  |  |  | endoplasmic reticulum membrane (GO:0005789) | 2.05 | 1.1E-07 | 1.3E-05 |
|  |  | cell-substrate junction (GO:0030055) | 2.40 | 2.1E-04 | 8.9E-03 |  |  |  | nuclear outer membrane-endoplasmic reticulum membrane network (GO:0042175) | 2.04 | 5.7E-08 | 7.0E-06 |
|  |  | focal adhesion (GO:0005925) | 2.38 | 4.3E-04 | 1.7E-02 |  |  |  | endoplasmic reticulum subcompartment (GO:0098827) | 2.04 | 9.8E-08 | 1.1E-05 |
|  |  | myofibril (GO:0030016) | 2.37 | 7.4E-05 | 4.0E-03 |  |  |  | transcription regulator complex (GO:0005667) | 1.9 | 2.1E-05 | 1.3E-03 |
|  |  |  |  |  |  |  |  |  |  |  |  |  |
| **Up-regulation at pH*_e_* 5.9** | | |  |  |  |  | **Down-regulation at pH*_e_* 5.9** | | |  |  |  |
|  | ***GO biological process complete*** | |  |  |  |  |  | ***GO biological process complete*** | |  |  |  |
|  |  | regulation of acrosomal vesicle exocytosis (GO:2000367) | 27.65 | 7.8E-04 | 4.0E-02 |  |  |  | alkaloid metabolic process (GO:0009820) | 19.48 | 1.8E-04 | 1.7E-02 |
|  |  | nitrobenzene metabolic process (GO:0018916) | 15.80 | 4.0E-04 | 2.4E-02 |  |  |  | positive regulation of transcription from RNA polymerase II promoter in response to endoplasmic reticulum stress (GO:1990440) | 17.04 | 7.3E-06 | 1.2E-03 |
|  |  | detoxification of nitrogen compound (GO:0051410) | 13.83 | 5.8E-04 | 3.2E-02 |  |  |  | positive regulation of endoplasmic reticulum unfolded protein response (GO:1900103) | 13.63 | 5.2E-04 | 4.1E-02 |
|  |  | cellular detoxification of nitrogen compound (GO:0070458) | 13.83 | 5.8E-04 | 3.2E-02 |  |  |  | positive regulation of transcription from RNA polymerase II promoter in response to stress (GO:0036003) | 11.36 | 4.6E-05 | 5.4E-03 |
|  |  | cellular response to interleukin-15 (GO:0071350) | 13.83 | 1.2E-04 | 8.9E-03 |  |  |  | serine family amino acid biosynthetic process (GO:0009070) | 10.65 | 2.6E-04 | 2.3E-02 |
|  |  | interleukin-15-mediated signaling pathway (GO:0035723) | 13.83 | 1.2E-04 | 8.8E-03 |  |  |  | regulation of DNA-templated transcription in response to stress (GO:0043620) | 10.22 | 1.9E-08 | 9.5E-06 |
|  |  | germinal center formation (GO:0002467) | 12.57 | 1.6E-04 | 1.2E-02 |  |  |  | regulation of transcription from RNA polymerase II promoter in response to stress (GO:0043618) | 9.74 | 4.3E-07 | 1.0E-04 |
|  |  | response to interleukin-15 (GO:0070672) | 12.57 | 1.6E-04 | 1.2E-02 |  |  |  | endoplasmic reticulum unfolded protein response (GO:0030968) | 8.36 | 3.7E-08 | 1.7E-05 |
|  |  | hepoxilin biosynthetic process (GO:0051122) | 9.22 | 5.1E-04 | 3.0E-02 |  |  |  | intrinsic apoptotic signaling pathway in response to endoplasmic reticulum stress (GO:0070059) | 7.30 | 1.2E-05 | 1.8E-03 |
|  |  | hepoxilin metabolic process (GO:0051121) | 9.22 | 5.1E-04 | 2.9E-02 |  |  |  | cellular response to unfolded protein (GO:0034620) | 7.23 | 5.4E-08 | 2.1E-05 |
|  |  |  |  |  |  |  |  |  |  |  |  |  |
|  | ***GO molecular function complete*** | |  |  |  |  |  | ***GO molecular function complete*** | |  |  |  |
|  |  | excitatory extracellular ligand-gated monoatomic ion channel activity (GO:0005231) | 5.67 | 2.00E-04 | 3.57E-02 |  |  |  | disulfide oxidoreductase activity (GO:0015036) | 7.02 | 1.4E-04 | 4.0E-02 |
|  |  | heparin binding (GO:0008201) | 2.80 | 1.86E-04 | 3.45E-02 |  |  |  | organic anion transmembrane transporter activity (GO:0008514) | 2.89 | 3.3E-05 | 1.6E-02 |
|  |  | sulfur compound binding (GO:1901681) | 2.68 | 4.93E-06 | 2.05E-03 |  |  |  | secondary active transmembrane transporter activity (GO:0015291) | 2.59 | 9.9E-05 | 2.9E-02 |
|  |  | cell adhesion molecule binding (GO:0050839) | 2.51 | 2.73E-05 | 7.20E-03 |  |  |  | active transmembrane transporter activity (GO:0022804) | 2.28 | 9.1E-05 | 2.9E-02 |
|  |  | cytokine receptor binding (GO:0005126) | 2.36 | 8.76E-05 | 1.99E-02 |  |  |  | protein domain specific binding (GO:0019904) | 1.94 | 4.1E-05 | 1.9E-02 |
|  |  | actin binding (GO:0003779) | 2.04 | 2.26E-04 | 3.78E-02 |  |  |  | enzyme binding (GO:0019899) | 1.57 | 3.7E-06 | 4.6E-03 |
|  |  | protein homodimerization activity (GO:0042803) | 1.90 | 1.13E-05 | 3.55E-03 |  |  |  | identical protein binding (GO:0042802) | 1.49 | 4.7E-05 | 2.0E-02 |
|  |  | signaling receptor binding (GO:0005102) | 1.75 | 7.00E-08 | 5.01E-05 |  |  |  | protein binding (GO:0005515) | 1.31 | 1.0E-11 | 5.1E-08 |
|  |  | protein dimerization activity (GO:0046983) | 1.75 | 1.27E-05 | 3.73E-03 |  |  |  | ion binding (GO:0043167) | 1.30 | 3.0E-05 | 2.1E-02 |
|  |  | transition metal ion binding (GO:0046914) | 1.73 | 5.26E-05 | 1.25E-02 |  |  |  | organic cyclic compound binding (GO:0097159) | 1.30 | 2.7E-05 | 2.2E-02 |
|  |  |  |  |  |  |  |  |  |  |  |  |  |
|  | ***GO cellular component complete*** | |  |  |  |  |  | ***GO cellular component complete*** | |  |  |  |
|  |  | integrin complex (GO:0008305) | 5.87 | 4.2E-04 | 2.4E-02 |  |  |  | endoplasmic reticulum chaperone complex (GO:0034663) | 19.88 | 5.5E-07 | 7.3E-05 |
|  |  | sarcoplasmic reticulum (GO:0016529) | 3.95 | 2.4E-04 | 1.6E-02 |  |  |  | smooth endoplasmic reticulum (GO:0005790) | 8.52 | 1.4E-05 | 1.4E-03 |
|  |  | basement membrane (GO:0005604) | 3.37 | 1.7E-04 | 1.2E-02 |  |  |  | endoplasmic reticulum-Golgi intermediate compartment (GO:0005793) | 5.61 | 2.1E-06 | 2.4E-04 |
|  |  | Z disc (GO:0030018) | 3.33 | 6.8E-05 | 5.0E-03 |  |  |  | endoplasmic reticulum protein-containing complex (GO:0140534) | 5.09 | 1.7E-08 | 2.4E-06 |
|  |  | sarcoplasm (GO:0016528) | 3.29 | 5.9E-04 | 3.3E-02 |  |  |  | endoplasmic reticulum lumen (GO:0005788) | 4.17 | 1.4E-04 | 1.3E-02 |
|  |  | I band (GO:0031674) | 3.20 | 6.3E-05 | 4.9E-03 |  |  |  | endoplasmic reticulum membrane (GO:0005789) | 2.61 | 3.0E-09 | 4.9E-07 |
|  |  | sarcomere (GO:0030017) | 3.01 | 1.3E-05 | 1.3E-03 |  |  |  | nuclear outer membrane-endoplasmic reticulum membrane network (GO:0042175) | 2.59 | 1.7E-09 | 3.3E-07 |
|  |  | collagen-containing extracellular matrix (GO:0062023) | 3.00 | 1.3E-09 | 4.2E-07 |  |  |  | endoplasmic reticulum subcompartment (GO:0098827) | 2.56 | 4.8E-09 | 7.3E-07 |
|  |  | extracellular matrix (GO:0031012) | 2.86 | 4.9E-11 | 2.5E-08 |  |  |  | basolateral plasma membrane (GO:0016323) | 2.53 | 2.6E-04 | 2.2E-02 |
|  |  | contractile fiber (GO:0043292) | 2.86 | 8.2E-06 | 8.5E-04 |  |  |  | basal plasma membrane (GO:0009925) | 2.35 | 5.5E-04 | 4.4E-02 |

*FDR: Benjamini-Hochberg False Discovery Rate
