# Supplementary material for: Clinical significance of acidic extracellular microenvironment modulated genes
Source: Front Oncol. 2024 Sep 20;14:1380679. doi: 10.3389/fonc.2024.1380679 (PMC11449683; doi:10.3389/fonc.2024.1380679)
Supplement: Supplementary file 5 [file Table3.docx]

| **Table S3. PANTHER GO analysis.** | | | | | | | | | | | | |
| --- | --- | --- | --- | --- | --- | --- | --- | --- | --- | --- | --- | --- |
|  |  | **Category** | **Fold  enrichment** | **P-value** | **FDR*** |  |  |  | **Category** | **Fold  enrichment** | **P-value** | **FDR*** |
| **Up-regulation at pH*_e_* 6.8** | | |  |  |  | **Down-regulation at pH*_e_* 6.8** | | | |  |  |  |
|  | ***PANTHER GO-Slim Biological Process*** | |  |  |  |  |  | ***PANTHER GO-Slim Biological Process*** | |  |  |  |
|  |  | defense response to virus (GO:0051607) | 4.80 | 1.5E-04 | 1.9E-02 |  |  |  | epithelial cilium movement involved in extracellular fluid movement (GO:0003351) | 19.51 | 2.1E-03 | 4.8E-02 |
|  |  | response to virus (GO:0009615) | 4.80 | 1.5E-04 | 1.8E-02 |  |  |  | endoplasmic reticulum unfolded protein response (GO:0030968) | 8.36 | 8.5E-06 | 6.7E-04 |
|  |  | glutathione metabolic process (GO:0006749) | 4.55 | 4.4E-04 | 2.7E-02 |  |  |  | response to unfolded protein (GO:0006986) | 7.63 | 1.5E-05 | 9.4E-04 |
|  |  | regulation of mitotic cell cycle phase transition (GO:1901990) | 3.32 | 1.1E-03 | 4.7E-02 |  |  |  | cellular response to unfolded protein (GO:0034620) | 7.63 | 1.5E-05 | 9.1E-04 |
|  |  | axon guidance (GO:0007411) | 3.12 | 1.1E-04 | 2.3E-02 |  |  |  | cellular response to topologically incorrect protein (GO:0035967) | 6.31 | 7.8E-06 | 6.6E-04 |
|  |  | neuron projection guidance (GO:0097485) | 3.12 | 1.1E-04 | 2.1E-02 |  |  |  | response to topologically incorrect protein (GO:0035966) | 6.31 | 7.8E-06 | 6.3E-04 |
|  |  | axonogenesis (GO:0007409) | 2.84 | 1.3E-04 | 2.2E-02 |  |  |  | response to endoplasmic reticulum stress (GO:0034976) | 5.28 | 1.8E-09 | 1.0E-06 |
|  |  | extracellular matrix organization (GO:0030198) | 2.82 | 1.4E-04 | 2.1E-02 |  |  |  | negative regulation of MAPK cascade (GO:0043409) | 4.55 | 1.8E-03 | 4.7E-02 |
|  |  | external encapsulating structure organization (GO:0045229) | 2.82 | 1.4E-04 | 1.9E-02 |  |  |  | negative regulation of protein kinase activity (GO:0006469) | 3.90 | 2.1E-03 | 4.9E-02 |
|  |  | extracellular structure organization (GO:0043062) | 2.78 | 1.7E-04 | 1.7E-02 |  |  |  | alpha-amino acid biosynthetic process (GO:1901607) | 3.90 | 2.1E-03 | 4.9E-02 |
|  |  |  |  |  |  |  |  |  |  |  |  |  |
|  | ***PANTHER GO-Slim Molecular Function*** | |  |  |  |  |  | ***PANTHER GO-Slim Molecular Function*** | |  |  |  |
|  |  | glutathione transferase activity (GO:0004364) | 5.61 | 1.2E-04 | 6.9E-03 |  |  |  | neutral L-amino acid transmembrane transporter activity (GO:0015175) | 5.02 | 2.3E-04 | 5.5E-03 |
|  |  | endopeptidase activity (GO:0004175) | 2.16 | 3.8E-04 | 1.8E-02 |  |  |  | amide transmembrane transporter activity (GO:0042887) | 4.55 | 1.8E-03 | 3.4E-02 |
|  |  | phosphoric ester hydrolase activity (GO:0042578) | 2.07 | 6.2E-04 | 2.6E-02 |  |  |  | polyubiquitin modification-dependent protein binding (GO:0031593) | 4.40 | 2.2E-03 | 3.9E-02 |
|  |  | enzyme regulator activity (GO:0030234) | 1.82 | 5.6E-05 | 3.7E-03 |  |  |  | L-amino acid transmembrane transporter activity (GO:0015179) | 4.37 | 3.4E-05 | 1.4E-03 |
|  |  | hydrolase activity, acting on ester bonds (GO:0016788) | 1.70 | 7.2E-04 | 2.9E-02 |  |  |  | amino acid transmembrane transporter activity (GO:0015171) | 3.62 | 1.8E-04 | 4.5E-03 |
|  |  | molecular function regulator activity (GO:0098772) | 1.70 | 1.0E-05 | 1.0E-03 |  |  |  | carboxylic acid transmembrane transporter activity (GO:0046943) | 2.69 | 7.1E-04 | 1.6E-02 |
|  |  | hydrolase activity (GO:0016787) | 1.63 | 3.6E-07 | 1.1E-04 |  |  |  | organic acid transmembrane transporter activity (GO:0005342) | 2.67 | 7.7E-04 | 1.6E-02 |
|  |  | protein binding (GO:0005515) | 1.44 | 1.4E-06 | 2.1E-04 |  |  |  | organic anion transmembrane transporter activity (GO:0008514) | 2.50 | 5.3E-04 | 1.2E-02 |
|  |  | catalytic activity (GO:0003824) | 1.33 | 6.7E-07 | 1.3E-04 |  |  |  | catalytic activity, acting on RNA (GO:0140098) | 2.34 | 1.1E-04 | 3.3E-03 |
|  |  |  |  |  |  |  |  |  | phosphoric ester hydrolase activity (GO:0042578) | 1.94 | 2.3E-03 | 4.0E-02 |
|  |  |  |  |  |  |  |  |  |  |  |  |  |
|  | ***PANTHER GO-Slim Cellular Component*** | |  |  |  |  |  | ***PANTHER GO-Slim Cellular Component*** | |  |  |  |
|  |  | extracellular matrix (GO:0031012) | 2.18 | 7.2E-05 | 8.9E-03 |  |  |  | endoplasmic reticulum-Golgi intermediate compartment (GO:0005793) | 6.10 | 2.6E-05 | 1.3E-03 |
|  |  | external encapsulating structure (GO:0030312) | 2.18 | 7.2E-05 | 7.1E-03 |  |  |  | coated vesicle (GO:0030135) | 2.94 | 7.0E-04 | 2.0E-02 |
|  |  | cell periphery (GO:0071944) | 1.29 | 4.0E-04 | 3.3E-02 |  |  |  | nuclear outer membrane-endoplasmic reticulum membrane network (GO:0042175) | 2.66 | 1.1E-05 | 8.6E-04 |
|  |  | cellular_component (GO:0005575) | 1.11 | 8.3E-06 | 2.0E-03 |  |  |  | endoplasmic reticulum membrane (GO:0005789) | 2.62 | 3.0E-05 | 1.3E-03 |
|  |  | cellular anatomical entity (GO:0110165) | 1.11 | 2.3E-05 | 3.8E-03 |  |  |  | endoplasmic reticulum subcompartment (GO:0098827) | 2.53 | 4.1E-05 | 1.7E-03 |
|  |  | Unclassified (UNCLASSIFIED) | 0.85 | 8.3E-06 | 4.1E-03 |  |  |  | organelle subcompartment (GO:0031984) | 1.98 | 7.7E-04 | 2.1E-02 |
|  |  | ribonucleoprotein complex (GO:1990904) | 0.35 | 6.1E-04 | 4.3E-02 |  |  |  | endoplasmic reticulum (GO:0005783) | 1.94 | 1.9E-05 | 1.1E-03 |
|  |  |  |  |  |  |  |  |  | endomembrane system (GO:0012505) | 1.64 | 1.0E-06 | 1.2E-04 |
|  |  |  |  |  |  |  |  |  | organelle membrane (GO:0031090) | 1.63 | 5.5E-04 | 1.8E-02 |
|  |  |  |  |  |  |  |  |  | cytoplasm (GO:0005737) | 1.35 | 3.5E-10 | 8.5E-08 |
|  |  |  |  |  |  |  |  |  |  |  |  |  |
|  | ***PANTHER Protein Class*** | |  |  |  |  | ***PANTHER Protein Class*** | | |  |  |  |
|  |  | Hsp70 family chaperone (PC00027) | 7.20 | 1.6E-03 | 2.9E-02 |  |  |  | basic leucine zipper transcription factor (PC00056) | 5.63 | 3.4E-06 | 1.7E-04 |
|  |  | actin or actin-binding cytoskeletal protein (PC00041) | 2.20 | 1.4E-04 | 4.4E-03 |  |  |  | aminoacyl-tRNA synthetase (PC00047) | 5.23 | 3.4E-05 | 1.3E-03 |
|  |  | G-protein modulator (PC00022) | 2.04 | 9.1E-04 | 1.8E-02 |  |  |  | translational protein (PC00263) | 1.92 | 6.7E-04 | 1.3E-02 |
|  |  | hydrolase (PC00121) | 1.79 | 3.0E-04 | 7.5E-03 |  |  |  | scaffold/adaptor protein (PC00226) | 1.64 | 3.7E-04 | 1.0E-02 |
|  |  | protein-binding activity modulator (PC00095) | 1.55 | 7.7E-04 | 1.7E-02 |  |  |  | DNA-binding transcription factor (PC00218) | 1.41 | 8.4E-04 | 1.5E-02 |
|  |  | metabolite interconversion enzyme (PC00262) | 1.37 | 1.4E-04 | 3.9E-03 |  |  |  | gene-specific transcriptional regulator (PC00264) | 1.36 | 2.3E-03 | 3.4E-02 |
|  |  | protein class (PC00000) | 1.08 | 5.4E-05 | 2.1E-03 |  |  |  | metabolite interconversion enzyme (PC00262) | 1.31 | 1.5E-03 | 2.5E-02 |
|  |  | Unclassified (UNCLASSIFIED) | 0.82 | 5.4E-05 | 2.7E-03 |  |  |  | protein class (PC00000) | 1.07 | 4.4E-04 | 1.1E-02 |
|  |  | G-protein coupled receptor (PC00021) | 0.51 | 1.7E-03 | 2.8E-02 |  |  |  | Unclassified (UNCLASSIFIED) | 0.84 | 4.4E-04 | 9.7E-03 |
|  |  | transmembrane signal receptor (PC00197) | 0.51 | 1.8E-09 | 3.5E-07 |  |  |  | transmembrane signal receptor (PC00197) | 0.39 | 5.0E-14 | 9.8E-12 |
|  |  |  |  |  |  |  |  |  |  |  |  |  |
|  | ***PANTHER Pathways*** | |  |  |  |  |  | ***PANTHER Pathways*** | |  |  |  |
|  |  | Unclassified (UNCLASSIFIED) | 0.94 | 8.0E-07 | 1.3E-04 |  |  |  | Unclassified (UNCLASSIFIED) | 0.95 | 5.2E-05 | 8.3E-03 |
|  |  |  |  |  |  |  |  |  |  |  |  |  |
|  | ***Reactome pathways*** | |  |  |  |  |  | ***Reactome pathways*** | |  |  |  |
|  |  | Cholesterol biosynthesis (R-MMU-191273) | 6.93 | 1.1E-05 | 3.1E-03 |  |  |  | Cargo concentration in the ER (R-MMU-5694530) | 5.91 | 3.2E-05 | 7.9E-03 |
|  |  | *O*-glycosylation of TSR domain-containing proteins (R-MMU-5173214) | 5.28 | 3.3E-05 | 8.1E-03 |  |  |  | COPII-mediated vesicle transport (R-MMU-204005) | 4.02 | 3.9E-05 | 8.4E-03 |
|  |  | Glutathione conjugation (R-MMU-156590) | 5.06 | 1.0E-04 | 1.9E-02 |  |  |  | ER to Golgi Anterograde Transport (R-MMU-199977) | 3.43 | 3.6E-07 | 3.1E-04 |
|  |  | Metabolism of steroids (R-MMU-8957322) | 3.39 | 2.1E-06 | 1.2E-03 |  |  |  | COPI-mediated anterograde transport (R-MMU-6807878) | 3.29 | 9.5E-05 | 1.7E-02 |
|  |  | Transmission across Chemical Synapses (R-MMU-112315) | 2.65 | 9.1E-06 | 3.1E-03 |  |  |  | Transport to the Golgi and subsequent modification (R-MMU-948021) | 2.85 | 7.5E-06 | 2.6E-03 |
|  |  | Neuronal System (R-MMU-112316) | 2.27 | 5.0E-06 | 2.1E-03 |  |  |  | Asparagine N-linked glycosylation (R-MMU-446203) | 2.66 | 9.0E-07 | 5.2E-04 |
|  |  | Extracellular matrix organization (R-MMU-1474244) | 2.23 | 1.6E-04 | 2.8E-02 |  |  |  | Membrane Trafficking (R-MMU-199991) | 1.98 | 4.1E-06 | 1.8E-03 |
|  |  | Metabolism of lipids (R-MMU-556833) | 1.77 | 9.3E-05 | 2.0E-02 |  |  |  | Transport of small molecules (R-MMU-382551) | 1.77 | 6.7E-05 | 1.3E-02 |
|  |  | Metabolism (R-MMU-1430728) | 1.65 | 3.7E-09 | 3.2E-06 |  |  |  | Vesicle-mediated transport (R-MMU-5653656) | 1.71 | 1.1E-04 | 1.8E-02 |
|  |  | Unclassified (UNCLASSIFIED) | 0.77 | 2.4E-19 | 4.1E-16 |  |  |  | Post-translational protein modification (R-MMU-597592) | 1.50 | 1.4E-04 | 2.0E-02 |
|  |  |  |  |  |  |  |  |  |  |  |  |  |
| **Up-regulation at pH*_e_* 5.9** | | |  |  |  |  | **Down-regulation at pH*_e_* 5.9** | | |  |  |  |
|  | ***PANTHER GO-Slim Biological Process*** | |  |  |  |  |  | ***PANTHER GO-Slim Biological Process*** | |  |  |  |
|  |  | Wnt signaling pathway (GO:0016055) | 4.79 | 2.5E-04 | 3.7E-02 |  |  |  | endoplasmic reticulum unfolded protein response (GO:0030968) | 12.98 | 1.1E-06 | 5.8E-04 |
|  |  | cell surface receptor signaling pathway involved in cell-cell signaling (GO:1905114) | 4.69 | 1.3E-04 | 2.7E-02 |  |  |  | response to unfolded protein (GO:0006986) | 11.85 | 1.8E-06 | 8.1E-04 |
|  |  | cell-cell signaling by wnt (GO:0198738) | 4.61 | 3.2E-04 | 3.7E-02 |  |  |  | cellular response to unfolded protein (GO:0034620) | 11.85 | 1.8E-06 | 6.8E-04 |
|  |  | cellular response to cytokine stimulus (GO:0071345) | 3.28 | 2.2E-04 | 3.7E-02 |  |  |  | cellular response to topologically incorrect protein (GO:0035967) | 9.02 | 2.8E-06 | 8.8E-04 |
|  |  | response to cytokine (GO:0034097) | 3.22 | 1.6E-04 | 2.9E-02 |  |  |  | response to topologically incorrect protein (GO:0035966) | 9.02 | 2.8E-06 | 7.7E-04 |
|  |  | neuron differentiation (GO:0030182) | 2.29 | 4.7E-04 | 4.7E-02 |  |  |  | response to endoplasmic reticulum stress (GO:0034976) | 7.22 | 7.0E-10 | 1.5E-06 |
|  |  | generation of neurons (GO:0048699) | 2.28 | 3.4E-04 | 3.8E-02 |  |  |  | alpha-amino acid biosynthetic process (GO:1901607) | 5.96 | 3.5E-04 | 4.0E-02 |
|  |  | cell adhesion (GO:0007155) | 2.25 | 8.2E-05 | 1.8E-02 |  |  |  | amino acid biosynthetic process (GO:0008652) | 5.68 | 4.5E-04 | 5.0E-02 |
|  |  | cell projection organization (GO:0030030) | 2.09 | 2.7E-04 | 3.5E-02 |  |  |  | carboxylic acid biosynthetic process (GO:0046394) | 3.75 | 1.8E-04 | 3.0E-02 |
|  |  | regulation of molecular function (GO:0065009) | 2.09 | 1.2E-05 | 5.1E-03 |  |  |  | organic acid biosynthetic process (GO:0016053) | 3.75 | 1.8E-04 | 2.8E-02 |
|  |  |  |  |  |  |  |  |  |  |  |  |  |
|  | ***PANTHER GO-Slim Molecular Function*** | |  |  |  |  |  | ***PANTHER GO-Slim Molecular Function*** | |  |  |  |
|  |  | phospholipid transporter activity (GO:0005548) | 6.64 | 6.2E-04 | 3.1E-02 |  |  |  | active transmembrane transporter activity (GO:0022804) | 2.74 | 2.2E-04 | 2.6E-02 |
|  |  | glutathione transferase activity (GO:0004364) | 6.45 | 2.5E-04 | 1.9E-02 |  |  |  | catalytic activity (GO:0003824) | 1.34 | 1.5E-04 | 2.2E-02 |
|  |  | integrin binding (GO:0005178) | 4.02 | 1.5E-03 | 5.6E-02 |  |  |  | signaling receptor activity (GO:0038023) | 0.44 | 1.2E-06 | 3.6E-04 |
|  |  | ligand-gated channel activity (GO:0022834) | 3.16 | 8.1E-04 | 3.7E-02 |  |  |  | molecular transducer activity (GO:0060089) | 0.44 | 1.2E-06 | 2.4E-04 |
|  |  | ligand-gated monoatomic ion channel activity (GO:0015276) | 3.16 | 8.1E-04 | 3.4E-02 |  |  |  | transmembrane signaling receptor activity (GO:0004888) | 0.38 | 2.1E-07 | 1.3E-04 |
|  |  | G protein-coupled receptor binding (GO:0001664) | 2.77 | 1.5E-03 | 5.4E-02 |  |  |  |  |  |  |  |
|  |  | actin binding (GO:0003779) | 2.66 | 1.1E-04 | 1.1E-02 |  |  |  |  |  |  |  |
|  |  | signaling receptor binding (GO:0005102) | 2.07 | 6.5E-06 | 7.7E-04 |  |  |  |  |  |  |  |
|  |  | enzyme regulator activity (GO:0030234) | 1.96 | 1.1E-04 | 9.4E-03 |  |  |  |  |  |  |  |
|  |  | molecular function regulator activity (GO:0098772) | 1.90 | 3.8E-06 | 5.7E-04 |  |  |  |  |  |  |  |
|  |  |  |  |  |  |  |  |  |  |  |  |  |
|  | ***PANTHER GO-Slim Cellular Component*** | |  |  |  |  |  | ***PANTHER GO-Slim Cellular Component*** | |  |  |  |
|  |  | collagen-containing extracellular matrix (GO:0062023) | 3.30 | 1.6E-05 | 2.6E-03 |  |  |  | endoplasmic reticulum (GO:0005783) | 2.29 | 1.4E-05 | 6.8E-03 |
|  |  | extracellular matrix (GO:0031012) | 2.95 | 2.4E-07 | 1.2E-04 |  |  |  |  |  |  |  |
|  |  | external encapsulating structure (GO:0030312) | 2.95 | 2.4E-07 | 5.9E-05 |  |  |  |  |  |  |  |
|  |  | receptor complex (GO:0043235) | 2.75 | 6.9E-05 | 6.8E-03 |  |  |  |  |  |  |  |
|  |  | cell periphery (GO:0071944) | 1.41 | 3.1E-05 | 3.8E-03 |  |  |  |  |  |  |  |
|  |  | cellular anatomical entity (GO:0110165) | 1.11 | 3.6E-04 | 2.2E-02 |  |  |  |  |  |  |  |
|  |  | cellular_component (GO:0005575) | 1.11 | 3.4E-04 | 2.4E-02 |  |  |  |  |  |  |  |
|  |  | Unclassified (UNCLASSIFIED) | 0.85 | 3.4E-04 | 2.8E-02 |  |  |  |  |  |  |  |
|  |  |  |  |  |  |  |  |  |  |  |  |  |
|  |  |  |  |  |  |  |  |  |  |  |  |  |
|  |  |  |  |  |  |  |  |  |  |  |  |  |
|  | ***PANTHER Protein Class*** | |  |  |  |  |  | ***PANTHER Protein Class*** | |  |  |  |
|  |  | ligand-gated ion channel (PC00141) | 4.29 | 5.1E-04 | 1.7E-02 |  |  |  | deaminase (PC00088) | 9.74 | 1.5E-03 | 3.2E-02 |
|  |  | guanyl-nucleotide exchange factor (PC00113) | 3.13 | 5.5E-04 | 1.5E-02 |  |  |  | extracellular matrix protein (PC00102) | 2.97 | 7.8E-04 | 1.9E-02 |
|  |  | G-protein modulator (PC00022) | 2.47 | 1.9E-04 | 9.1E-03 |  |  |  | scaffold/adaptor protein (PC00226) | 1.83 | 5.2E-04 | 1.7E-02 |
|  |  | actin or actin-binding cytoskeletal protein (PC00041) | 2.20 | 1.5E-03 | 2.8E-02 |  |  |  | metabolite interconversion enzyme (PC00262) | 1.49 | 1.8E-04 | 1.2E-02 |
|  |  | cell adhesion molecule (PC00069) | 2.17 | 7.1E-04 | 1.8E-02 |  |  |  | protein class (PC00000) | 1.09 | 2.4E-04 | 1.2E-02 |
|  |  | ion channel (PC00133) | 2.11 | 1.4E-03 | 2.7E-02 |  |  |  | Unclassified (UNCLASSIFIED) | 0.78 | 2.4E-04 | 9.3E-03 |
|  |  | intercellular signal molecule (PC00207) | 1.98 | 8.4E-04 | 1.8E-02 |  |  |  | transmembrane signal receptor (PC00197) | 0.38 | 9.4E-09 | 1.9E-06 |
|  |  | cytoskeletal protein (PC00085) | 1.71 | 2.2E-03 | 3.3E-02 |  |  |  | defense/immunity protein (PC00090) | 0.27 | 2.1E-05 | 2.0E-03 |
|  |  | transporter (PC00227) | 1.64 | 2.7E-04 | 1.1E-02 |  |  |  | immunoglobulin (PC00123) | 0.10 | 6.1E-04 | 1.7E-02 |
|  |  | protein-binding activity modulator (PC00095) | 1.61 | 1.8E-03 | 2.9E-02 |  |  |  |  |  |  |  |
|  |  |  |  |  |  |  |  |  |  |  |  |  |
|  | ***PANTHER Pathways*** | |  |  |  |  |  | ***PANTHER Pathways*** | |  |  |  |
|  |  | Unclassified (UNCLASSIFIED) | 0.92 | 4.6E-08 | 7.5E-06 |  |  |  | No statistically significant results. |  |  |  |
|  |  |  |  |  |  |  |  |  |  |  |  |  |
|  | *Reactome pathways* | |  |  |  |  |  | *Reactome pathways* | |  |  |  |
|  |  | Glutathione conjugation (R-MMU-156590) | 5.98 | 1.5E-04 | 5.0E-02 |  |  |  | Transport of small molecules (R-MMU-382551) | 2.26 | 1.5E-06 | 1.3E-03 |
|  |  | Biological oxidations (R-MMU-211859) | 2.78 | 6.1E-05 | 2.6E-02 |  |  |  | Unclassified (UNCLASSIFIED) | 0.83 | 2.5E-07 | 4.3E-04 |
|  |  | Cytokine Signaling in Immune system (R-MMU-1280215) | 2.26 | 3.2E-05 | 1.9E-02 |  |  |  |  |  |  |  |
|  |  | Metabolism (R-MMU-1430728) | 1.63 | 2.0E-06 | 1.7E-03 |  |  |  |  |  |  |  |
|  |  | Signal Transduction (R-MMU-162582) | 1.43 | 1.7E-04 | 5.0E-02 |  |  |  |  |  |  |  |
|  |  | Unclassified (UNCLASSIFIED) | 0.80 | 4.6E-11 | 8.0E-08 |  |  |  |  |  |  |  |

*FDR: Benjamini-Hochberg False Discovery Rate
